# Clinical Updates in Cardiac Pacing—The Future Is Bright

**DOI:** 10.3390/jcm11216376

**Published:** 2022-10-28

**Authors:** Bert Vandenberk, Brennan A. Ballantyne, Derek S. Chew

**Affiliations:** 1 Department of Cardiovascular Sciences, KU Leuven, 3010 Leuven, Belgium; 2 Department of Cardiac Sciences, Libin Cardiovascular Institute, Cumming School of Medicine, University of Calgary, Calgary, AB T2N 1N4, Canada

The history of cardiac pacing has been defined by many innovation milestones starting in the early 1960s. Cardiac pacing technology evolved from asynchronous pacing to physiologic dual chamber pacing in the early 1990s [1]. Unlike our contemporary approach, the original definition of physiologic pacing referred to the preservation of atrioventricular (AV) synchrony, as this was associated with improved patient tolerance and a lower risk of atrial arrhythmias [2]. In the subsequent decade, cardiac resynchronization therapy (CRT) was introduced, which remains a cornerstone therapy in patients with symptomatic heart failure in combination with guideline-directed medical therapy. Additionally, patients with AV block who require a high percentage of ventricular pacing benefit from CRT implantation [3]. Recent advances that will continue to push the field forward into the future include leadless pacemakers and conduction system pacing (CSP).

Despite its immense clinical impact, approximately 30% of CRT patients will not experience clinical or echocardiographic benefits. Research is ongoing to find high-yield approaches to decrease the proportion of CRT non-responders. Targeted LV lead delivery during CRT implant has been investigated in TARGET and STARTER trials and has shown improvement in short-term left ventricular remodeling, as well as long-term mortality [4,5,6]. Further, cardiac magnetic-resonance-imaging-guided delivery of both the left and right ventricular leads has shown promising results, with data from a randomized clinical trial being expected shortly [7]. A better understanding of the underlying pathophysiology of reverse left ventricular remodeling might further improve patient selection. There is a continuous search for refined selection criteria, including an assessment of mechanical dyssynchrony. Recently, several mechanical dyssynchrony parameters were associated with a reduction in mitral valve regurgitation [8]. However, CSP, referring to both His bundle pacing as left bundle branch area pacing, may challenge the future of CRT. Recent studies have shown a larger improvement in left ventricular ejection fraction with CSP, as well as improved clinical outcomes in patients eligible for CRT [9,10]. In patients with non-ischemic cardiomyopathy and left bundle branch block, left bundle branch area pacing was associated with a larger improvement in left ventricular ejection fraction after 6 months of follow-up (mean difference: 5.6%; 95% CI: 0.3–10.9; *p* = 0.039) and in a greater reduction in left ventricular end-systolic volumes (−24.97 mL; 95% CI: −49.58 to −0.36 mL) [10].

Another revolution in cardiac pacing was the introduction of leadless pacemakers. Currently, two leadless pacemakers have been approved by the United States Food and Drug Administration: the Micra TPS (Medtronic, Minneapolis, MN, USA) and the AVEIR (Abbott, Chicago, IL, USA) [11]. In the LEADLESS II study, published in 2015, 526 patients were prospectively enrolled and implanted with the first leadless pacemaker, Nanostim (Abbott, Chicago, IL, USA) [12]. The implant was successful in 95.8% of patients; the serious adverse event rate was 6.7% after 6 months of follow-up. Eventually, Nanostim was recalled due to premature battery depletion and reported spontaneous separation of the docking/retrieval button. In 2016, the Micra IDE study was published [13]. A total of 725 patients were implanted, with an implant success rate of 99.2% and a major complication rate of 4.0% [13]. Given the limitations of single-chamber pacing with loss of atrioventricular synchrony, Micra TPS evolved to the Micra AV using atrial mechanical sensing [14]. The Nanostim has been upgraded and returned to the market as the AVEIR leadless pacemaker with an ongoing clinical trial, the AVEIR DR i2i study, using atrial leadless pacemakers and dedication communication technology [15,16]. The experience with leadless pacing is growing and has identified patient populations who may derive particular benefit from leadless pacemakers, such as patients with difficult vascular access, prior valve interventions, and high infection risk [17,18,19,20]. A direct comparison between leadless and conventional transvenous pacemakers is still lacking and may identify additional patient populations who could be eligible for leadless pacemaker implantation. Further, animal experiments with leadless CRT options, the WiSe-CRT system, are ongoing and may facilitate CRT in difficult cases [21].

Contributions exploring the impact of these novel cardiac-pacing technologies by experts in the field will be collected as part of the Special Issue entitled “Clinical Updates in Cardiac Pacing” by the *Journal of Clinical Medicine*. These contributions can provide valuable insights into recent developments and may lead to significant research advances in cardiac pacing. 
